# On the autonomy of the concept of disease in psychiatry

**DOI:** 10.3389/fpsyg.2013.00457

**Published:** 2013-07-19

**Authors:** Thomas Schramme

**Affiliations:** Department of Philosophy, Hamburg UniversityHamburg, Germany

**Keywords:** concept of mental illness, mind-body problem, identity theory, reductionism, eliminativism, Szasz, Kendell, biological psychiatry

## Abstract

Does the reference to a mental realm in using the notion of mental disorder lead to a dilemma that consists in either implying a Cartesian account of the mind-body relation or in the need to give up a notion of *mental* disorder in its own right? Many psychiatrists seem to believe that denying substance dualism requires a purely neurophysiological stance for explaining mental disorder. However, this conviction is based on a limited awareness of the philosophical debate on the mind-body problem. This article discusses the reasonableness of the concept of mental disorder in relation to reductionist and eliminativist strategies in the philosophy of mind. It is concluded that we need a psychological level of explanation that cannot be reduced to neurophysiological findings in order to make sense of mental disorder.

For some time, especially in the 60s and 70s of the twentieth century, psychiatry was under pressure because it did not seem capable of showing that mental illness actually exists. Can there really be such a thing as a “disease of the mind”? At the time skeptics such as Thomas Szasz (1974) wrote against the “myth of mental illness.” The emphasis of this debate lay for a long time in the scrutiny of the associated norms, i.e., the question whether one can differentiate between sick or healthy mental phenomena. One still visible effect of this debate, which has more or less reached its conclusion, is the avoidance of the term “disease” in the official nomenclature of psychiatric medicine. Nowadays we speak of *disorders*, not diseases, for example in the Diagnostic and Statistic Manual of Mental Disorders (DSM), or in the International Statistical Classification of Diseases and Related Health Problems (ICD), in which the psychiatric classification is also described as “mental and behavioral disorders.” One cannot help feeling that the attempt had been to avoid a definition that is already all too close to somatic medicine, which would have required a corresponding “hard” validation of the respective categories, or at least a set of explanations of the nosological units in these classification systems. Because contemporary psychiatry barely meets this idea, one could get out of this whole affair with a “weaker” term^1^.

The downside of this difficulty is visible in the likewise wide-spread somatization of psychopathology, which—put briefly—is the identification of mental disorders as diseases of the brain, a practice that has a long tradition in the history of psychiatry. All efforts are undertaken to establish psychopathological phenomena as “real,” precisely because many psychiatrists share the view that psychiatry has to be scientifically demonstrable and ought to put in place generally testable criteria for pathological conditions, and because it is somewhat embarrassing for them to separate “disorder” from the disease concept in such a way. Because the real is in turn considered to be one and the same as the observable, from this point on the material disorder of the brain counted as what ideally should be proven. The move had some initial plausibility, in that one more aspect of the original skepticism toward the concept of disease in psychiatry could be undermined. This doubt was fed by the supposedly non-existing location of the disturbance: the spirit or the psyche. How can one rationally assume scientifically valid criteria of pathology when the impaired or damaged object apparently does not exist, or at the very least cannot be affected by disorders? The answer lay in tossing away significant reference to the psyche, the mind, or to mental objects, and talking mainly about neurophysiology, the nervous system, and the brain; for instance when schizophrenia was regarded as a disorder of dopamine and serotonin levels. The somatization could therefore solve both problems of psychiatry: its ostensibly poor scientific grounding and the supposed lack of reality of its phenomena. One astonishing result of this development lay furthermore in the fact that leading proponents of psychiatry now claim—just as their strongest critics once had—that mental illnesses do not actually exist, because only brain disease exists. From a philosophical perspective such a conclusion seems unreasonable^2^.

The proponents of the concept of disease in psychiatry, however, seem to be left indeed in an uncomfortable dilemma: If they emphasize the bodily manifestation of mental diseases, then they save the analogy to somatic disease and therefore the medical terminology. At the same time they stand to lose through this strategy the uniqueness of mental disease, which is reduced to somatic disease. If one wants on the other hand to keep the distinctive manner of speaking of *mental* disease (or disorder), then this is apparently only possible when one at the same time postulates a sphere of the mind that is distinct from the body. This strategy, again, seems to lead to a mind-body-duality in the mode that has become unpopular in philosophy. The supposed dilemma for proponents of the concept of mental disease therefore consists in the choice between the Scylla of reduction and the Charybdis of dualism. “Psychiatry is left with two seeming alternatives: either to say that personal, psychological, and emotional disorders are really states of the body, objective features of brain-tissue, the organism-under-stress, the genes or what have you; or else to deny that such disorders are illnesses at all” (Sedgwick, 1973).

As already indicated, there are authors, especially on the side of psychiatry, who are ready to solve this dilemma by consistent somatization and consequentially abandoning the concept of mental disease. One prominent example for this is Robert Kendell (1993, 3), a well-known British psychiatrist: “[…] it follows that there is, strictly speaking, no such thing as disease of the mind or mental disorder and that Griesinger was right—mental illnesses are diseases of the brain, or at least involve disordered brain function—because all mental events are accompanied by and dependent on events in the brain. (Thomas Szasz was also right; mental illness is a myth, though not for the reasons he believed.).” A surprising alliance between biological psychiatrists and skeptics has been formed; in the end both positions are ready to give up the concept of mental illness.

The task of this article is therefore to search for compelling arguments in favor of a distinctive concept of mental illness. As already indicated, I cast doubt on the presented dilemmatic structure as a defense of this concept. Rather, I shall try to show that there are well-reasoned positions somewhere between the two horns of the dilemma. The question—can we see “*mental* illness” as an autonomous concept without relying upon unpleasant theoretical premises?—is, as I have said, based on an important philosophical issue: the mind-body problem. This problem can be formulated as follows: Are there really mental phenomena, and if so, how can they be explained and how are they connected with physical phenomena? The first part of this question may sound rather strange, since what are we more sure of than that mental states such as pain, wishes and beliefs exist?^3^ In the course of this investigation it will become clearer—so I hope at least—why questioning the existence of mental phenomena is not as strange as it seems. The second part of the mind-body problem, the question of the nature of mental phenomena and their relation to physical states, has of late, with the study of the brain and the nervous system, received a strong empirical orientation and even a new twist. The idea that the nervous system and especially the brain are the basis of mental states is nowadays no longer seriously disputed. For this reason the mind-body problem is sometimes reformulated as the “mind-brain problem.” The assumption that mental phenomena are based upon physiological processes suggested itself after the observation of people with brain injuries. Since the introduction of new imaging techniques to study the brain's physiological processes, this theory has been strengthened and refined. Today we can identify connections between regions in the brain and specific mental capabilities, even if definitive relationships between them are not yet possible. And ultimately we are still far away from being able to formulate a generally accepted solution to the mind-body problem.

In this article I will first briefly focus on Thomas Szasz, the main critic of the concept of mental illness. Here I will examine especially his arguments that are related to the mind-body problem^4^. Second, I will apply reductionist and eliminative theories in the philosophy of mind in order to find out whether they might rule out the concept of mental illness.

## The sceptical arguments of thomas szasz

Like no other theorist, Szasz has dealt with the concept of disease in psychiatry in a very intensive way, and has attempted to demonstrate particularly the different disanologies between the concept of disease in somatic medicine and psychiatry. The concept of disease—as Szasz has put it in the argument that interests us—cannot be applied to mental phenomena, because the expressions “body” and “mind” belong to different logical categories. When we speak of mental *illness*, we are merely using the term as a metaphor.

In this argument Szasz relies upon the British philosopher Gilbert Ryle (1949), who, in his book *The Concept of Mind*, attacked the “official doctrine” of Cartesianism, in which every human being possesses both a body and a mind, which exist as independent entities. Ryle also called this position the “dogma of the ghost in the machine.” He wanted to refute this position by showing that it is based on a so-called category mistake. Szasz uses Ryle's approach in order to demonstrate that the “official doctrine” of psychiatry commits an identical category mistake, in virtue of assuming that there is both bodily illness and mental illness. The mistake consists in the claim that the mind can, just like the body, be affected by illness.

Ryle explains his conception of a category mistake by way of several examples. Suppose that a student comes to Oxford for the first time. There she is given a tour of the many colleges, libraries, and administrative offices. After the tour she says: “I now know the individual colleges and where the books are kept, and I have also seen where the administrators work. But now I want to be shown the university.” Naturally this wish cannot be fulfilled, because colleges, libraries, etc. *are* the university. There is no other point of interest that is called “university.” Apparently the student does not know that “university” belongs to a category separate from “New College,” “Bodleian Library,” etc. It would be a similar mistake to ask, after one had been shown the functions of the defender, the forward, and the goalkeeper etc., whose function it was to contribute the team spirit in a football team.

The mistake of Cartesianism is given, according to Ryle, in such a mixing of categories, in that “mind” is classified in the same logical category as “body.” Only in this way could it be claimed that a human being has both a mind and a body. But one could only combine expressions in linguistically correct conjunctions when they belong to the same category. Therefore, one cannot, from a linguistic point of view, state, for example that one has seen New College, Bodleian Library, *and* Oxford University.

Ryle does not claim that the mind cannot be said to exist as a matter of principle. But if this claim is made, one would need another meaning of “exist” than in the assertion that bodies exist. Because mind and body belong to different categories, it cannot be reasonably claimed that mind *and* body exist in the same way. Nor can it be expressed in a logically compelling way that *either* the mind *or* the body exists, because this disjunction is just as inadmissible when expressing different categories. To say, “Either I have visited the New College, the Bodleian Library etc. or seen the university” is patently absurd.

At this point Szasz (1974) wants to show that *because* “body” and “mind” belong to different logical categories, *in principle* there can be no such thing as a mental illness. This theory and its basis—the category mistake argument—are important for Szasz's work and are mentioned again and again, even when there is no direct relation to Ryle, such as when Szasz says that an illness can only affect the body.

As previously mentioned, Ryle's category mistake argument is directed at the “official doctrine” that had prevailed after Descartes and that postulated two distinct entities: the body and the mind. The special feature of Ryle's argument as compared to other critiques of dualism is his claim that asking about the relationship between mind and body is already by itself non-sensical (Ryle, 1949, 23). But even if this view is correct, the category mistake argument alone does not support Szasz's theory, because the mere suggestion that “mind” and “body” belong to different categories does not allow the conclusion that a mental illness cannot exist. The argument does not express whether mental phenomena exist or not, but rather casts doubt on Cartesianism.

Moreover, illness does not exist independent from organisms. If one abandons the notion of an independent existence for illness, the idea of actual mental illness is no longer implausible, because we need not claim that the mind *has* an illness, but rather, as one could say for example, a disorder of mental capacities—however specified—*is* a mental illness. In order to make this claim one would not have to postulate a separation between mind and body. Substance dualism is not required for the maintenance of the concept of mental illness. Therefore, Szasz cannot show the absurdity of this concept in principle through casting doubt on the Cartesian separation of mind and body.

## The mind-body problem

There are many different theories about the relationship between mental and physiological states. These theories range from strict separation of the two spheres to their identification, or lead all the way to the claim that the mind-body problem is principally unsolvable. This situation and the enormous scale that the respective literature has since reached make it necessary to restrict this examination to a cursory treatment and to keep as strictly as possible to the issue at stake. For this reason I will largely restrict myself to negative statements in order to defend the concept of mental illness, and will attempt to show accordingly that the theories that doubt the explanatory independence of mental phenomena are not adequately justified to reject such an autonomy^5^. Positive criteria of such evaluation of theories would be their scientific adequacy—this spoke against Cartesian dualism—but also their philosophical plausibility, of course. Still, I cannot deal with all of the many contributions in this area of research.

Theories of the relation between mind and body that lead to a questioning of the mental disease concept are largely of two kinds: Either they are *reductive*—they lead to an explanation of mental phenomena through reference to physiological states; or else they are *eliminative*—they relegate mental phenomena to the sphere of myth that is, dispute the very existence of mental states. I naturally do not want to claim that the respective authors actually want to discard the notion of mental illness. Ultimately their theories are aimed at a different question. But it is possible to scrutinize the independence of the mental disease concept on the basis of such theories and in doing so to ultimately scrutinize the identity of psychology and psychiatry. And I think furthermore that implicit or explicit theories about the connection between mind and body can have an impact on psychiatry's research focuses, its methods of treatment, and its classifications.

From this point on I will take it for granted that the problems of substance dualism, such as those exemplified in Descartes, are already well-known. In the following I will first analyse the reductive theories on the mind-body problem. Then I will examine the eliminative theories. The result will show that the rejection of an independent concept of mental illness does not succeed. A conceptualization of mental disease by referring to mental states is possible without having to advocate an awkward dualism. Nevertheless, we should not go so far as to generally repudiate somatic approaches in psychiatry, but rather emphasize their one-sidedness and their need to be complemented. Psychiatry should be neither “mindless” nor “brainless”.^6^

## Identity-theory

The mind-body identity theory makes reference to mental states, which underlie our actions, and therefore it does not reduce them to observable behavior, as behaviorism had done previously, ultimately leading to a theoretical dead end. Identity theory permits explanations of behavior that appeal to desires, beliefs, pain, etc., and seems in this regard adequate to work as a basis for an explication of the mental illness concept. However, the theory posits the ontological identity of mental states and neurophysiological states. In this way identity theory rules out substance-dualistic assumptions. Mental states are identical to physiological states of the nervous system and the brain. There are not two different substances, only one, namely matter. The mental does not have any properties that go beyond the physiological^7^. One often cited example for this is the identity of pain with the stimulation of C-fibers.

Identity theory is superior to behaviorism not only thanks to its appeal to inner states, but also through its ability to acknowledge different mental phenomena in cases of identical observable behaviors. For identical behaviors can have many different underlying mental states, which are themselves identical to specific neurophysiological states. Therefore, for instance the same behavioral abnormality could in one case be accompanied by a neurophysiological irregularity that is completely missing in another case. On the basis of such a theory this suggests searching for the boundaries between mental normality and illness on a neurophysiological level, because on this level different mental states manifest themselves. The first step to a theory about mental illness as a brain illness is therefore fulfilled. Identity theory also solves Descartes's problem of explaining mental causation, for instance the causation of the action of going to the fridge by a desire to drink and a belief that there is a drink in the fridge. On the basis of substance dualism such a straightforward explanation becomes quite difficult to achieve, because mental phenomena are regarded as non-material in Cartesianism, and non-material things lack the force to cause anything physical. However, if mental states are identical to neurophysiological states, then there is no need to postulate an obscure non-mental causality. Mental states can cause material changes to the body in virtue of their material existence.

We should clarify what the identity theorists mean by “identity.” J. J. C. Smart (1959, 171), one of the prominent proponents of identity theory, speaks of “strict identity” and uses the identity of lightning and electric discharge as explanatory example. The thesis is stronger than a mere claim to a correlation between brain states and mental phenomena. This type of identity is often explained using Leibniz's law of the identity of indiscernibles: X is strictly identical to Y if they are indiscernible from one another, i.e., if every property of X is also a property of Y, and vice versa. In the present case of an identity claim, this means that there are no properties of the mental that are not also properties of the nervous system. Hence there is no unique property of the mental.

The proposition of identity theorists is not tantamount to the claim that statements about mental and neurophysiological states have the same meaning, and are therefore synonymous. The identity theory merely claims that the state being referred to is one and the same. We can elucidate this distinction with the following example: The Evening Star is identical with the Morning Star (both have the same properties of the planet Venus, and so refer to the same object), although the terms have different meanings, because there are “different modes of presentation” at hand (Frege, 1892).

Smart (1959) emphasizes the difference between synonymity and identity in order to avoid one obvious objection to identity theory. For there's room to claim that mental and physical states are not identical, because someone who has no idea of neurophysiology can nonetheless refer to his mental condition. If the two states were identical, so this argument goes, then we could substitute the proposition in a statement such as “I know that my foot hurts” with the proposition “my brain is in state X” while retaining the truth value. Since this clearly does not work, the states cannot be identical. This only shows, however, that statements about mental states have a different meaning than statements about physical states, not that they are not ontologically identical. Therefore, this kind of argumentation against identity theory is invalid.

In the distinction between meaning and reference there is also the implication that the postulated identity of mental and physical statements is informative (i.e., not *a priori*). In its differentiation between types of identity statements such as “a bachelor is an unmarried man,” “a square is an equilateral rectangle,” and the like, identity theory contains an assertion of a contingent, empirically verifiable fact. Mental states could also be identical with completely different states, but it has been established by science that they are identical with neurophysiological states.

Still, the critique of identity theory was not hard to come by. At this point two variations of objections can be distinguished^8^. Firstly, the neurophysiological side of the identity proposition has been the starting point for objections. Here the argument of “multiple realizability” is especially pertinent. In the case that indistinguishable mental states could each be realized through different brain states, they would not, contrary to the proposition of identity theory, be identical to specific neurophysiological states after all. Secondly, it is questionable whether mental states in their usual taxonomy can be rendered at all as identical with any underlying brain states. The terminology, with which we categories mental states as desires, hopes, etc., has been around long before anyone ever had any insight into brain processes, and it seems therefore unlikely that clear equivalents can be found.

The identity theorists postulate a strict identity of mental states and neurophysiological states. The proposition is that indistinguishable mental phenomena each correspond to the same physiological states. But, as the objection goes, it is not only conceivable, but even extremely likely that many animals experience states of consciousness such as for example pain. Yet the nervous system and brains of many animals vary widely from those of humans. Therefore, it is completely unlikely to ever be able to identify specific neurophysiological states with mental states. A bird's feeling of pain, or that of a perhaps unknown creature, could be based on radically different physiological states. This is one possible way of formulating the argument of “multiple realizability.”

The objection itself was first advanced by Hilary Putman (1967). For strong forms of identity theory it has proven to be fatal, but not necessarily for weaker ones. Putnam supports identity theory with a strong proposition. He claims that the theory has to show, for example that a feeling of pain can always be identified with a specific neurophysiological state. If, however, different physical states can accompany the same mental state (feelings of pain), then this proposition falls apart. “Consider what the brain-state theorist has to do to make good his claims. He has to specify a physical-chemical state such that *any* organism (not just a mammal) is in pain if and only if (a) it possesses a brain of a suitable physical-chemical structure; and (b) its brain is in that physical-chemical state. This means that the physical-chemical state in question must be a possible state of a mammalian brain, a reptilian brain, a mollusc's brain (octopuses are mollusca, and certainly feel pain), etc. At the same time, it must *not* be a possible (physically possible) state of the brain of any physically possible creature that cannot feel pain” (Putman, 1967, 53).

The philosophers that support identity theory have only inadequately explained what they understand as mental states. It would be, for example, conceivable not to simply identify unspecified feelings of pain with one neurophysiological state, but rather “sharp” pain, “dull” pain, etc. It would be equally possible, naturally, to realize the classification of species-specific mental states. Then human pain could be identified with the neurophysiological state X, octopus pain with mollusc state Y, etc. Such species-oriented reduction seems to weaken Putman's argument. But even if we restrict ourselves to examples of mental phenomena in humans, it remains unlikely to be able to carry out identification at a higher level of mental states. Consider, for example, a desire to travel to London. This desire realizes itself clearly within an entire web of other mental states: for instance the belief that London lies in England; the belief that London is beautiful; the knowledge that one has an important appointment there. Even given all that we know about the brain, it appears hopeless ever to be able to match such a state one-for-one with a neurophysiological state. The brain (and also the respective mental state) is simply too complex to be able to ascribe such identities^9^.

There is still the alternative of weakening statements of identity by making the classification of mental states more finely granulated. Hence it could be the case, for example that we can identify a specific neurophysiological state with the mental state “sensing an orange in a veiled and darkened room.” Yet here it would be unclear what advantage would come of this kind of reduction. The more that identity theory specifies the types of mental states that they want to identify, the more they lose its original advantage, namely its simplicity. But it does not necessarily follow from the lack of plausibility of such type-identification that mental phenomena are not, on particular levels, after all identical with physical events. This is the proposition of the so-called token-identity. This theory attributes the identity of every single mental event (token), for example the belief of person X at time *t* that it will rain today, to a single neurophysiological event. This is admittedly an extremely weak identity thesis, but it guarantees the maintenance of a non-dualistic position. Still, it apparently prevents any *reasonable* reduction of the explanation of mental states to physiological states, and is therefore inadequate as an argument against the autonomy of the concept of mental illness.

There is a yet another way to understand type-identity: If every realization of a type of mental states could be subsumed to a class of neurophysiological states, then the argument of multiple realization would be defeated. Suppose the belief that Berlin is the capital of Germany would be realized in the respective neurophysiological states N1, N2, N3, etc. Due to this assumption multiple realizability would be safeguarded, because the belief would not always be identical to one and the same neurophysiological state. If, however, N1, N2, N3, etc. could be subsumed to a class, then a kind of type-identity would be saved (Rosenthal, 1994, 351; Hannan, 1994, 21f.).

Maybe we could identify particular mental illnesses with distinct classes of neurophysiological states, in the sense that all realizations of a mental illness would fall under a neurophysiological class. The following is an example: Suppose that schizophrenia in person X were realized in neurophysiological state N(X), in person Y in state N(Y), etc. Now it appears that N(X), N(Y), etc. all belong to a distinct class S. Then it would evidently be possible to reduce the explanation for schizophrenia to the class S. Whether we can show that I cannot say, but the chances seem (theoretically) not so bad^10^. On the other hand the question remains whether through S we have really explained and reduced the mental phenomena-type schizophrenia completely. This is arguably not the case. One important reason to me seems to be the following: To subsume such mental phenomena as “schizophrenia” assumes that they have in common a particular property, namely that they are cases of disorders of “normal” *mental* states. Even if it were indeed the case that S underlies all these states, the mere reference to S does not explain the pathology of the schizophrenic realizations (see also Margolis, 1991). We only show that there is a *correlation* between the mental states and a type of neurophysiological states. The claim that all instances of S have the property of realizing a *disordered* neurophysiological process is only possible on the psychological level of explanation. The fact that specific mental phenomena count as a mental illness cannot therefore be explained exclusively through brain physiology. In this respect I take the assertion that mental illnesses are brain *illnesses* (or diseases, for that matter) to be truncated. The assertion conceals that being ill is explained and only recognizable on the psychological level. A psychological explanation of mental illness is in this regard autonomous in relation to physiological attempts at explanation.

Even if all counterarguments up to now are not sufficient for a complete repudiation of identity theory, can the theory ever show itself to be true? The second of the objections to be presented here focuses on the mental side of the identity thesis in replying to the question introduced, i.e., the question about the possibility of reducing the mental to the physical level^11^. Mental states are normally grouped into so-called propositional attitudes (for example desires, beliefs, hopes, which all have propositional content) and sensations (pain, sensual perception, etc.). We use this classification in order to make actions comprehensible. If we see someone running in the train station, then we understand this behavior in that we ascribe to the person certain beliefs—for example that the train is about to leave—and desires—that he wants to board the train before it leaves. We can even make our own actions comprehensible with these categories, such as in the following example: “I took my hand away because I suddenly felt a sharp pain.” At the same time there are certain principles that guide our explanations, such as, for instance, if a person would carry out action A when she has the chance, because she has the desire X and believes that A will achieve X. Together these categories and principles amount to an apparently useful basis for the explanation and prediction of behavior. This is not an especially complicated psychological theory that requires its own field of study, but rather an accumulation of concepts and rules, which are regularly used on an everyday basis. This tool has been given the short-hand name “folk-psychology”^12^. Already for hundreds of years—and long before anyone ever knew about brain processes—this has been used by people, at least in a similar form, in order to interpret their behaviors. Yet identity theory claims that there is a one-to-one correspondence between neurophysiological processes—any knowledge about which we have only ever gathered in the last few decades—and the taxonomy of folk-psychology. It seems more than unlikely, however, that categories that originated long before our knowledge of the brain and nervous system can be precisely identified with corresponding neurophysiological types of processes. It would be just about as likely as a correspondence between the disease taxonomy of Paracelsus and the newest insights into physiological processes in the human body.

## Eliminative materialism

The results of this examination up to now suggest a certain skepticism toward reductive theories in philosophy of the mind. To be sure, mental states are not on the one hand non-physiological states, since they are apparently realized through physiological processes in the brain. On the other hand the world of the mental is not entirely physiologically explicable. “The desire to drink a beer causes him to go into the pub.” “The detective followed the murderer because he believed he wanted to hide the weapon of the crime.” It is questionable how such complex situations could be described meaningfully on a purely physical level. In this regard we have levels of explanation that are independent and irreducible. Folk-psychology works on this level. But are its explanations correct? Proponents of Eliminative Materialism (EM) answer this question in the negative^13^. Going further, they actually come to the radical conclusion that these states, which we and other creatures are ascribed to within the framework of folk-psychology, do not exist. There are no desires, beliefs, fears, etc. This thesis contains the eliminative side of EM. Propositional attitudes (and even states of experience like feelings of pain, here depending on what EM exactly sees as elements of folk-psychology)^14^ are removed of their ontological legitimation, whereby they are relegated to the sphere of myth.

This is a truly radical proposition, and I will try to illustrate why it is not as unintelligible as it may at first seem. Certainly it should be clear that—if EM is right—it would have equally radical consequences for our conception of mental illness. If the sphere of the mental, in the way that we describe and explain it every day, is non-existent in the strictest sense, then this would really revolutionize its conceptualization as well as the categorization of single types of illnesses. Whether the mental disease concept in its own independent framework would also be lost depends on which level of explanation is chosen for the corresponding phenomena. If there were a (future) scientific psychology that would then replace the eliminated folk psychology, then talk of *mental* disease would—in my opinion—still be warranted^15^. However, because the main supporters of EM want to see folk psychology eliminated to the benefit of a (future) *neuro*physiology, it is questionable whether there could still be *mental* illnesses in the strictest sense^16^.

The discussion concerning EM's persuasiveness is therefore linked primarily to the status of folk psychology. Ironically the latter's irreducibility becomes a symptom of its superfluity. Because mental states are based on physical states, a neurophysiological explanation should support the folk-psychological explanation. That was also the claim of identity theory. Now it has been shown, however, that explanations on the level of folk psychology cannot be reduced to a neurophysiological level. And precisely because this irreducibility relation exists, it can be shown that folk psychology and neurophysiology are incommensurable. Temperature could be successfully reduced to molecular kinetic energy, and therefore can continue to be seen as existing. Conversely, no one could find a scientific explanation for witches that were commensurable with superstition, and so witches count as non-existing. The more we learn about the functionality of the brain the clearer it becomes that there are practically no equivocations to be made from the folk-psychologically explained mental states to neurophysiological levels. But when two approaches of explanation are incompatible, then it is to be taken that at least one is false, in which case it should be discarded. The claim of EM is naturally that folk psychology is false and therefore has to disappear. To this degree mental states go the way of witches: They are expelled from the scientific ontology.

In short, EM consists of two premises and one conclusion (cf. Stich, 1996, 4). Premise 1: Folk psychology is a theory. Premise 2: Folk psychology is false. Conclusion: The mental states postulated by folk psychology do not exist and can play no role in any future explanation of behavior.

Which objections are addressed toward EM?^17^ Its first premise pertains to the form of folk psychology: Does it really represent a theory? This claim is essential for EM's proposition, for otherwise it would be questionable why we should drop folk psychology. The assumption of EM is that folk psychology aims to provide explanations and predictions of behavior, and therefore contains certain claims and supports certain principles, etc. If this were not the case, then it would not compete with a neurological explanation of the same issue, and would therefore not be an eligible candidate for elimination. It seems to me, on the basis of the already discussed need for a psychological perspective to establish distinct mental disorders that we cannot abandon the corresponding vocabulary and its related theoretical constructs.

The second premise of EM states that folk psychology as a theory is false, because it leaves many phenomena unexplained and is therefore incompatible with the underlying natural sciences: “(…) what we must say is that FP [folk psychology, TS] suffers explanatory failures on an epic scale that it has been stagnant for at least twenty-five centuries, and that its categories appear (so far) to be incommensurable with or orthogonal to the categories of the background physical science whose long term claim to explain human behavior seems undeniable. Any theory that meets this description must be allowed a serious candidate for outright elimination” (Churchland, 1981, 212).

Churchland sees shortcomings to the explanations of folk psychology in the areas of, e.g., creativity, intelligence, sleep, memory, sensory illusions, and—especially interesting for our analysis—in relation to mental illness. This suggestion of Churchland's is not easy to counter. It seems true that we can learn fairly little about mental illness through the help of folk-psychological conceptions and principles. But does folk psychology even have this aim? A theory can only be untrue in that area where it claims to have explanatory value. Otherwise it does not at all seek to compete with other theories such as neurophysiology or a “scientific” psychology.

Churchland's claim that folk psychology breaks down or fails as a theory apparently stems from the assumption that it does *compete* with neurophysiology. Then it would be an appropriate candidate for elimination. But this assumption is not straightforward. First the role and aim of folk psychology would have to be clarified. There is much to indicate that even when it is not all that successful as a theory, neither is it so false that it has to be eliminated, nor does it have to be replaced by a (future) neurophysiology. Even if it is replaced, it would be done so by a scientific theory that is situated on the same level of explanation—that is, a psychological one. Folk psychology works on a macro level, and neurophysiology on a micro level. In this regard the irreducibility of folk psychology does not necessarily lead to its incommensurability with neurophysiology. Each explains phenomena, but simply on two different levels of abstraction.

Psychiatric explanations of disease phenomena would not manage without psychological conceptions. Which role folk psychology will keep in the process is still undecided; but to eliminate it is neither necessary on theoretical grounds nor advisable on practical grounds, for it is through its coarse grain that in the end the psychological perspective facilitates our access to the mental world.

## Conclusion

The reductive and eliminative theories of the mind-body problem are not convincing. The reductive versions largely fail for the lack of correlation between types of mental and neurophysiological states. Identity theory seems to apply on the level of single mental events. Thereby, however, only an ontologically non-dualistic position is implied, and not at all the superfluity of mental terminology. On the contrary, mental illness is one of the very phenomena that would be unexplainable on an exclusively neurophysiological level, because here no explanation of a single event (token) is being sought, and because the ascription of pathology itself can only happen by taking the mental level into account. The eliminative position fails largely due to its ascription to unreasonably high expectations on the part of folk psychology. Sure it is unlikely that we can adequately explain mental illness with folk-psychological terminology alone. But this finding neither makes folk psychology as such superfluous nor excludes the possibility of a scientific and therefore *psychological* (and specifically psychiatric) explanation of mental illness.

The concept of mental illness was drawn into question on the basis of reductive and eliminative theories. As a consequence the rejection of these accounts leads to the possibility of an independent conceptualization of mental illness—so long as no further, more convincing objections are brought forward. To be sure, the difficulties of substance dualism prevent any possible explanation of mental illness to be completely independent of physiological knowledge. Mental and bodily phenomena do not belong to *principally* separate areas. In this regard both the general rejection and the one-sided restriction to brain-physiological explanations of mental illness fail. In brief, even if we have accepted that theories of mental illness are not allowed to be in conflict with the knowledge of neurophysiology, it does not follow that the single (let alone the best) explanation of mental illness can exist on a neurophysiological level.

Even if for many disorders of the mental apparatus no corresponding disorders of brain-physiological processes were found, the language of mental illness does not seem to me to have necessarily failed. The desire for “objective” signs of disease, as they are supposed to be in the brain or in observable behaviors, is understandable. Nevertheless, this is in the end a methodological problem that is posed for a future scientific study of mental illness. It should not lead to discounting any of the two—neurophysiological or mental—perspectives. Mental illness is not reducible to brain illness, even when mental phenomena have their basis in the brain.

The recent publication of the fifth version of the *Diagnostic and Statistical Manual of Mental Disorders* should cause occasion for the underlying philosophical aspects of the language of mental disorder to make itself clear within the psychiatric trade. The chairpersons of the working committee that put together the DSM-IV had still formulated the following misgivings: “There could arguably not be a worse term than *mental disorders* to describe the conditions classified in DSM-IV. *Mental* implies a mind-body dichotomy that is becoming increasingly outmoded (…)” (Frances, 1994, VIII). We should have expected that such mistaken misgivings—which are after all concerning the very foundations of psychiatry—would be resolved by now. A cursory look at current psychiatric publications, however, gives us cause to fear that the same error in reasoning will be made as before: “‘Mental’ implies a Cartesian view of the mind-body problem that minds and brains are separable and entirely distinct realms, an approach that is inconsistent with modern philosophical and neuroscientific views” (Stein et al., 2010, 1760) (^18^). In this respect we surely have to be skeptical that psychiatric thinking will have seriously progressed. Instead the cure will very likely be looked for in neurobiology. In this article I have attempted to show why psychiatry will not find here what it is looking for and that there is no need to look for a supposed cure, since the concept of mental illness is autonomous from somatic medicine. For philosophers this is no surprising realization; for many psychiatrists, however, the insight seems to remain closed off.

### Conflict of interest statement

The author declares that the research was conducted in the absence of any commercial or financial relationships that could be construed as a potential conflict of interest.

## Footnotes

^1^In this paper I use “disease,” “illness” and “disorder” synonymously.

^2^In this contribution I draw on considerations that were published first in German in my book Schramme (2000).

^3^This assumption in reference to the existence of the mental on the basis of its experiential aspects has also once again gained some weight recently in philosophy of psychiatry. To this end new phenomenological approaches, such as those from Fuchs (2004), Gallagher and Vaever (2004), or Ratcliffe (2008), have taken up the experiential attributes of mental disorders. From this perspective we could claim that the challenge of the mental by somatization is itself drawing upon a dualistic starting-point. In virtue of doubting distinct properties of the mental realm, somatization accordingly even results in a rejection of one aspect of human existence, i.e., the mental, which, however, cannot be separated from human corporeality. The contrasting holistic perspective becomes prominent in the concept of Leib (lived body), which accordingly plays an important role in the phenomenological tradition.

^4^A more extensive critical evaluation of Szasz's position, and of other critics of the concept of mental illness, can be found in Schramme (2004).

^5^Unfortunately there is still very little cooperation between philosophy and psychiatry regarding this specific issue as well as the underlying mind-body problem. The psychiatric literature is relatively unaffected by the elaborate debates in philosophy. In turn, a theory in philosophy will still seldom be examined against concrete examples, such as those found in psychiatry. It would be important to achieve stronger cooperation here, and on the whole I see this article to be an attempt at mediation. There are in the meantime some interesting publications that are headed in this direction. To highlight just a few: Wilkes (1988); Graham and Stephens (1994); Griffiths (1994); Northoff (1997); Ghaemi (2003); Radden (2004); Murphy (2006); Bolton (2008); Kendler and Parnas (2008); Graham and Stephens (2010), and Graham and Stephens (1994) founded periodical Philosophy, Psychiatry & Psychology; see also Schramme and Thome (2004).

^6^Eisenberg and Lipowski coined the terms “mindless” and “brainless” psychiatry (compare Sullivan (1990), p. 271).

^7^It is meant thereby that every mental state can in principle be fully explained exclusively in reference to neurophysiological processes. Compare for example Smart (1959) p. 54.

^8^Here I ignore a whole line of objections, which deal with so-called qualia that is, the specific, felt qualities of a mental state. These objections are not limited to the identity theory introduced here, but rather are in part turned in general against physicalism, disputing therefore that mental states possess exclusively physical attributes. The best known versions of these objections come from Nagel (1974) and Jackson (1982, 1986).

^9^Beckermann (1996, 6) points out that after injuries to the brain many mental processes of other parts of the brain, which previously were not involved, can be realized. To an extent this would be an intrapersonal variation of the argument of multiple realization. In my opinion the neuroplasticity of the brain is a general argument against the possibility of simple identification, which, however, I will ignore.

^10^Practically there are certainly significant problems, for in order to show that the single neurophysiological processes actually fall under a certain type one has to acquire a detailed knowledge of them. However, “The more precisely one wants to establish neural state N as the origin of a behavior, the greater does one change the brain and the overall situation of the test subject … ” (Tetens, 1992, p. 121). Tetens describes this as a “fuzziness relation” of neurobiology, and argues “Already for this reason naturalistic descriptions of the human being are not practically realizable alternatives to the mental-psychological descriptions.”

^11^It can be found for example in Churchland (1988 p. 27 f.) This objection is, as it will later become clear, not merely advanced against the plausibility of identity theory, but also serves as an argument for eliminative materialism.

^12^Sometimes common-sense-psychology is also being used, because the expression “folk psychology” has a pejorative connotation.

^13^The term “eliminative materialism” stems from Cornman (1968). It was already used earlier in the sixties by Feyerabend (1970) and Rorty (1965) in a similar form. The main supporters of a “modern” EM are Patricia and Paul Churchland.

^14^Sometimes only propositional attitudes are attributed to folk psychology. Hannan (1994, p. 45) points out that supporters of EM are often less critical of qualitative states. But Churchland (1994, (308) explicitly specifies pain for example as a component of folk psychology).

^15^Naturally I do not want to claim that the future psychology will manage without any reference to physiological and chemical processes in the brain. Already today the borders are fluid. The independence of the concept of mental illness would be kept alive in the maintenance of a genuine psychological terminology in this science. An attempt to discuss the theory of EM from a psychiatric point of view is undertaken by Harrison (1991).

^16^In this regard EM as such is not a threat to the mental illness concept (even if for today's existing classification of mental illnesses), but rather only in a certain version. Some arguments for why a future science of mental phenomena should not be exclusively located on a neural level can be found in Kitcher (1996). To be sure, it is not always clear from the Churchlands' writings whether they expect the elimination of psychology in general or of only folk psychology.

^17^One objection to EM that I do not find convincing, but will mention for the sake of thoroughness, is that it is self-defeating: The theory of EM contains the premise that beliefs, etc. do not exist. It is objected that one can only sensible formulate this premise, however, with recourse to the affected states themselves, for example beliefs. To me this objection seems to be begging the question, because it assumes the same manner of speaking that is put in question. An expression of the theses of EM may very well radically change, once folk psychology is eliminated. Hannan (1994, p. 62 ff.) puts more faith in this objection. For a defense against this charge see Churchland (1986, p. 397 f.); Churchland (1988, p. 48).

^18^This article was mentioned on the DSM-5 website as background for the planned revision of the definition of “mental disorder”: http://www.dsm5.org/proposedrevision/Pages/proposedrevision.aspx?rid=465# [Accessed 14. September 2012]. The reference has since been deleted from the website.
